# Unprofessional Behavior Among House Officers in Pakistan’s Public and Private Hospitals: Prevalence, Perceptions, and Participation

**DOI:** 10.7759/cureus.72461

**Published:** 2024-10-27

**Authors:** Sana Noor, Fatima Aslam, Azhar Ejaz, Aafia Malik, Hafsa Nasir, Nabila Awan, Imran Qadir Khattak, Mustaqeem Shah

**Affiliations:** 1 Community Medicine, Avicenna Medical and Dental College and Hospital, Lahore, PAK; 2 Medical Education, University of Health Sciences, Lahore, PAK; 3 General Surgery, Worcestershire Acute Hospital, National Health Service (NHS) Trust, Worcester, GBR; 4 Psychiatry, Herefordshire and Worcestershire Health and Care Trust, Worcester, GBR; 5 Surgery, Services Hospital, Lahore, PAK; 6 Medicine, Avicenna Medical and Dental College and Hospital, Lahore, PAK; 7 Internal Medicine, Hayatabad Medical Complex, Peshawar, PAK

**Keywords:** healthcare system, medical professionals, patients, professionalism, unprofessional behavior

## Abstract

Background: Professionalism in healthcare is crucial for maintaining patient trust and delivering high-quality care. Unprofessional behaviors among house officers (recent medical graduates undergoing training) raise concerns about their impact on healthcare outcomes. This study aims to assess the prevalence and perceptions of unprofessional behavior, as well as participation in it, among house officers in both public and private hospitals in Pakistan.

Methods: A cross-sectional survey was conducted among 212 house officers from seven hospitals across Pakistan. The hospitals included both public and private institutions: 57% were private (Avicenna Medical College, Rashid Latif Medical College, Amna Inayat Medical College, Nawaz Sharif Medical College) and 43% were public (Quaid-e-Azam Medical College, Gujranwala Medical College, Khyber Medical College). A pre-validated and pre-tested questionnaire was used, covering demographic data and 29 questions related to unprofessional behaviors, including perceptions, observations, and self-reported participation. Data were collected through an online platform and analyzed using IBM SPSS Statistics for Windows, version 22 (IBM Corp., Armonk, NY, USA). Descriptive statistics were used to summarize variables, and Chi-square tests for independence were applied to compare proportions between groups (graduates from private and public universities) for each variable. Associations between demographic factors and knowledge of professionalism were analyzed, with a significance level set at p ≤ 0.05.

Results: Out of 212 participants, almost all (99.1%, n=210) were familiar with the concept of professionalism, but over half (53.8%, n=114) had not completed a formal medical ethics course, while 52.4% (n=111) engaged in self-directed study. Although 78.3% (n=166) had completed additional professional ethics training, Chi-square analysis revealed no significant associations between ethics training and changes in unprofessional behaviors, nor between gender, department, or type of institution, and the likelihood of observing or participating in unprofessional behavior (p > 0.05). However, certain unprofessional behaviors were significantly associated with previous ethics training (p < 0.05).

Conclusion: While house officers demonstrated a strong awareness of professionalism, unprofessional behavior persisted regardless of gender or type of institution. These findings emphasize the need for enhanced ethical training and stricter monitoring to address and mitigate unprofessional behaviors in healthcare settings. Policymakers should consider mandatory integration of comprehensive ethical training in medical curricula to ensure better alignment between ethical knowledge and practice.

## Introduction

In healthcare, professionalism is fundamental in maintaining the integrity and trust that society places in the medical profession. It encompasses not only the conduct of healthcare providers but also the quality of the doctor-patient relationship [1,2]. Professionalism refers to the behavioral standards, ethical values, and commitment of individuals working within a particular profession. Medical professionalism, specifically, reflects the ability of healthcare professionals to organize, work efficiently, and deliver high-quality care while upholding the highest ethical standards [3].

Despite the critical importance of professionalism, there has been a concerning rise in unprofessional behaviors within the healthcare field [4]. This underscores the urgent need to emphasize professionalism, particularly in medical education, where future healthcare providers are shaped. The development of professional behavior in medical students is significantly influenced by their knowledge, perceptions, and attitudes during their training [5]. Studies have shown that when students perceive unprofessional behaviors as normative, they may adopt these practices themselves. One study highlighted that while there was a difference in perception of unprofessional behavior between junior and senior medical students, the rate of participation in such behavior showed no significant variation across these groups [6-8].

The relationship between professionalism, personality traits, and empathy in healthcare providers was explored in a cross-sectional study conducted at an Irish medical school. The study employed the Jefferson Scale of Empathy and the NEO Five-Factor Inventory (NEO-FFI) scale to measure these factors, finding that empathy and personality characteristics are independently linked to various components of professionalism [9].

A key element of effective patient-physician interaction is the proper introduction of medical professionals to patients and their families. However, a mixed-method study at Shahid Sadoughi University of Medical Sciences found that a significant proportion of medical students and residents failed to achieve this, contributing to unprofessional practices. The study reported high participation in unprofessional behaviors [10,11].

The qualitative aspect of the same study identified several factors contributing to unprofessionalism, including a lack of identity formation, insufficient recognition of responsibility, and the absence of mechanisms for reporting unprofessional conduct [12]. In striving to deliver the best possible healthcare, professionalism plays a pivotal role. Both patient and faculty perspectives are crucial in enhancing the quality of care. A qualitative study involving focus groups and in-depth interviews with patients and faculty members identified several key areas, including doctor-patient communication, reflective skills, time management, interprofessional relationships, and collegiality, as vital to professional development.

Objective

This cross-sectional study aimed to assess the prevalence and perceptions of unprofessional behavior, as well as participation in it, among house officers in both public and private hospitals in Pakistan.

## Materials and methods

Study design and ethical approval

The cross-sectional study was reviewed and approved by the Research Ethics Review Board (IRB) at Avicenna Medical College (IRB No: IRB-46/11/23/AVC; dated November 22, 2023). Informed consent was obtained from all participants, ensuring they understood the study's purpose, procedures, potential risks, and their right to withdraw at any time without penalty. The research utilized a quantitative methodology to obtain structured data from participants.

Participants

The target population for this study consisted of house officers employed in various public and private hospitals throughout Pakistan. The hospitals included both public and private institutions: 57% were private (Avicenna Medical College, Rashid Latif Medical College, Amna Inayat Medical College, Nawaz Sharif Medical College) and 43% were public (Quaid-e-Azam Medical College, Gujranwala Medical College, Khyber Medical College). A convenient sampling method was employed to include participants who were readily available and willing to participate. Inclusion criteria encompassed house officers with at least three months of clinical experience, while those not involved in direct patient care were excluded from the study.

Data collection

Data collection took place from November 2023 to August 2024. A structured, pre-validated, and pre-tested questionnaire was utilized for data collection, designed to be distributed through online Google Forms (Google, Mountain View, CA, USA) (see Appendices). This ensured accessibility and anonymity for participants.

Questionnaire design

The questionnaire was developed following a comprehensive review of the literature on professionalism and unprofessional behavior in healthcare, consisting of two primary sections. The first section gathered demographic information, including participants' age, gender, months of experience as a house officer, department, and type of medical institution (public or private). The second section focused on the assessment of unprofessional behavior, featuring 29 items that explored participants’ perceptions, observations, and personal involvement in unprofessional acts, including their participation in medical ethics courses and other professional ethics training, such as workshops or seminars on ethical decision-making, confidentiality, and maintaining professional boundaries.

Validity and reliability

The questionnaire underwent a rigorous validation process. The content validity was established through expert reviews, ensuring that all items accurately reflected the constructs being measured. The questionnaire was piloted with a sample of 30 house officers, yielding a Cronbach’s alpha coefficient of 0.83, indicating high internal consistency. Test-retest reliability was also assessed, demonstrating stability over time with a correlation coefficient of 0.85.

Quality assurance

To ensure data accuracy and reliability, the questionnaire was refined based on pilot testing feedback. The confidentiality of participant responses was strictly maintained, and no identifiable information was linked to the collected data.

Data analysis

Data were collected via Google Forms and subsequently exported to IBM SPSS Statistics for Windows, version 22 (IBM Corp., Armonk, NY, USA) for statistical analysis, where descriptive statistics were calculated to summarize demographic characteristics and responses related to unprofessional behavior. Specifically, means, frequencies, and percentages were determined for all relevant categorical variables, providing a comprehensive overview of the data. To explore the relationships between various demographic factors and unprofessional behaviors, Chi-square tests were conducted, with a p-value of ≤ 0.05 considered statistically significant. The analysis focused on three key variables: gender versus unprofessional behaviors, departments versus knowledge of professionalism, and graduating colleges versus knowledge of professionalism. The statistical analysis encompassed both descriptive statistics, which summarized demographic variables and observations of unprofessional behavior, and Chi-square tests which assessed associations between categorical variables.

## Results

**Figure 1:**
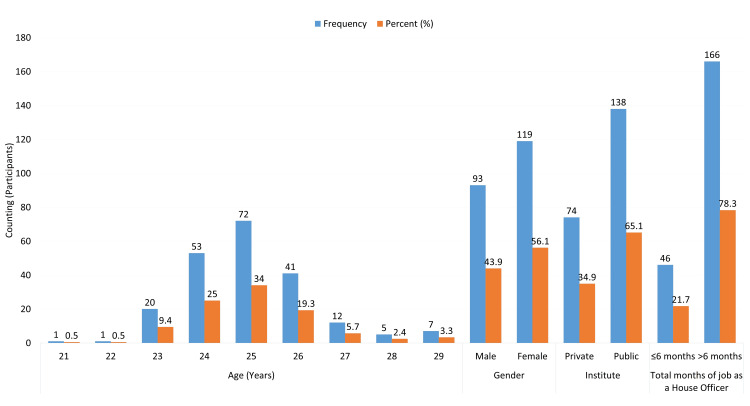
Demographic profile of house officers.

The demographic analysis of the study participants reveals that the majority of house officers are concentrated in the age group of 25 years, comprising 72 individuals (34%) (Figure 1). The participants had a mean age of 25.04±1.3 years. The age distribution also indicates that 53 (25%) are 24 years old, while the age group of 26 years includes 41 participants (19.3%). Younger age groups such as 21 (0.5%) and 22 (0.5%) years have minimal representation, with only one individual in each category. Conversely, older age groups show limited numbers, with 12 participants (5.7%) aged 27, five (2.4%) aged 28, and seven (3.3%) aged 29. Gender distribution shows a slightly higher representation of females, with 119 participants (56.1%) compared to 93 males (43.9%). Out of 212 house officers, 138 (65.1%) graduated from public medical institutes and 74 (34.9%) graduated from private medical institutes. In terms of experience, a significant majority of house officers, 166 individuals (78.3%), have worked for more than six months, while 46 participants (21.7%) have less than or equal to six months of experience. This data suggests a predominance of house officers aged 24-26 years, a notable female majority, and a higher level of experience among the participants.

**Figure 2:**
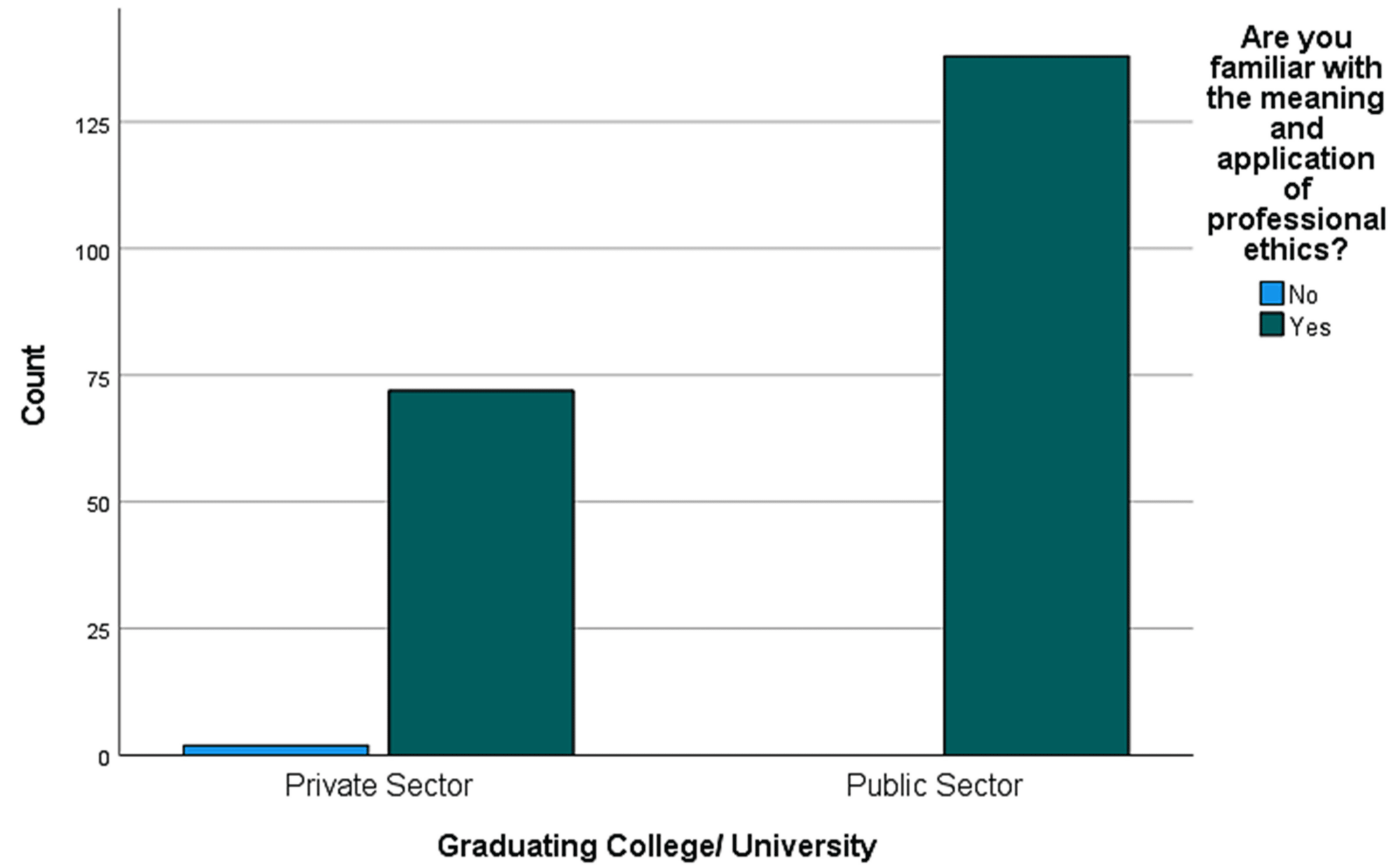
Familiarity with professionalism and significant engagement in ethics training among house officers. Graduating College/University refers to the completion of medical education at either a medical college or a medical university in Pakistan, both of which are primary institutions for medical training.

The result showed that the majority (99.1%) of the house officers were familiar with the meaning and application of professionalism (Figure 2). Among the house officers, 114 (53.8%) had passed a course in medical ethics, and 78.3% of house officers (n=212) had passed another professional ethics training course. More than half of the house officers agreed that they have self-directed study in the context of professionalism.

The major findings of the study revealed that most house officers had worked in a combination of medicine, surgery, and other departments (n=138, 65.1%; p=0.157) (Table 1). A larger proportion of participants graduated from public sector institutions (n=138, 65.1%) compared to private institutions (n=74, 34.9%; p=0.187). Hostelites made up the majority of the participants (n=145, 68.4%; p=0.224). Almost all house officers (210, 99.1%; p=0.494) were familiar with professional ethics, but over half had not passed a medical ethics course (n=114, 53.8%; p=0.048), while 111 house officers (n=52.4%; p=0.030) reported engaging in self-directed study on professionalism.

**Table 1:** Prevalence and statistical significance of unprofessional behaviors among house officers across various demographic and professional variables. * p-values less than 0.05 were significant.

Variable	Category	Frequency	Percent (%)	P-value
Departments in which you have worked up till now	Medicine & others	13	6.1	0.157
	Medicine & surgery	5	2.4	
	Medicine, surgery & others	138	65.1	
	Others	35	16.5	
	Surgery & others	21	9.9	
Graduating college/university	Private sector	74	34.9	0.187
	Public sector	138	65.1	
Residence	Day scholar	67	31.6	0.224
	Hostelite	145	68.4	
Familiar with professional ethics?	No	2	0.9	0.494
	Yes	210	99.1	
Passed a course in medical ethics before?	No	114	53.8	0.048*
	Yes	98	46.2	
Passed other professional ethics training course?	No	166	78.3	0.328
	Yes	46	21.7	
Self-directed study in professionalism?	No	101	47.6	0.030*
	Yes	111	52.4	
Lack of medical dignity (talking, dressing) with patients/colleagues?	Neither observed, nor participated	51	24.1	0.134
	Observed	128	60.4	
	Observed & participated	29	13.7	
	Participated	4	1.9	
Lack of respect for religious and cultural differences?	Neither observed, nor participated	114	53.8	0.034*
	Observed	79	37.3	
	Observed & participated	12	5.7	
	Participated	7	3.3	
Lack of self-assessment and refusal to accept critiques?	Neither observed, nor participated	64	30.2	0.028*
	Observed	126	59.4	
	Observed & participated	17	8	
	Participated	5	2.4	
Lack of commitment to continuous learning?	Neither observed, nor participated	65	30.7	0.366
	Observed	103	48.6	
	Observed & participated	37	17.5	
	Participated	7	3.3	
Lack of equity and fairness in serving patients?	Neither observed, nor participated	72	34	0.320
	Observed	108	50.9	
	Observed & participated	26	12.3	
	Participated	6	2.8	
Lack of acceptance of probable health risks for patient’s care?	Neither observed, nor participated	93	43.9	0.451
	Observed	84	39.6	
	Observed & participated	27	12.7	
	Participated	8	3.8	
Failure to comply with hospital regulations and policy?	Neither observed, nor participated	74	34.9	0.598
	Observed	83	39.2	
	Observed & participated	51	24.1	
	Participated	4	1.9	
Lack of bearing discomfort in responding to patients’ medical needs?	Neither observed, nor participated	71	33.5	0.045*
	Observed	101	47.6	
	Observed & participated	34	16	
	Participated	6	2.8	
Disregard of educational activities?	Neither observed, nor participated	45	21.2	0.724
	Observed	104	49.1	
	Observed & participated	59	27.8	
	Participated	4	1.9	
Denial of errors or mistakes?	Neither observed, nor participated	63	29.7	0.0097*
	Observed	116	54.7	
	Observed & participated	27	12.7	
	Participated	6	2.8	
Medical negligence in hospital duties?	Neither observed, nor participated	60	28.3	0.0001*
	Observed	120	56.6	
	Observed & participated	29	13.7	
	Participated	3	1.4	
Dishonest behavior in the workplace?	Neither observed, nor participated	67	31.6	0.0021*
	Observed	123	58	
	Observed & participated	16	7.5	
	Participated	6	2.8	
Lack of observance of discipline in medical work?	Neither observed, nor participated	45	21.2	0.0032*
	Observed	131	61.8	
	Observed & participated	33	15.6	
	Participated	3	1.4	
Lack of commitment to be available when “on call”?	Neither observed, nor participated	75	35.4	0.002*
	Observed	120	56.6	
	Observed & participated	12	5.7	
	Participated	5	2.4	
Prefer own interests to patient’s interests?	Neither observed, nor participated	87	41	0.444
	Observed	98	46.2	
	Observed & participated	23	10.8	
	Participated	4	1.9	
Not suggesting treatment options due to patient’s financial limitations?	Neither observed, nor participated	120	56.6	0.001*
	Observed	60	28.3	
	Observed & participated	28	13.2	
	Participated	4	1.9	
Lack of commitment to patient privacy?	Neither observed, nor participated	120	56.6	0.047*
	Observed	69	32.5	
	Observed & participated	17	8	
	Participated	6	2.8	
Failure to maintain professional boundary in relationships?	Neither observed, nor participated	109	51.4	0.049*
	Observed	84	39.6	
	Observed & participated	16	7.5	
	Participated	3	1.4	
Use of alcohol or drugs in the workplace?	Neither observed, nor participated	168	79.2	0.004*
	Observed	36	17	
	Observed & participated	5	2.4	
	Participated	3	1.4	

Specific unprofessional behaviors were significantly observed, including a lack of respect for religious and cultural differences (n=79, 37.3%; p=0.034), refusal to accept critiques (n=126, 59.4%; p=0.028), and lack of commitment to responding to patients’ medical needs (n=101, 47.6%; p=0.045). Denial of errors (n=116, 54.7%; p=0.0097), medical negligence (120, 56.6%; p=0.0001), and dishonest behavior in the workplace (n=123, 58%; p=0.0021) were also frequently observed. The use of alcohol or drugs in the workplace was noted by 36 house officers (17%; p=0.004), and failure to maintain professional boundaries was observed by 84 (39.6%; p=0.049).

A series of Chi-square tests were conducted to explore the relationships between various factors and unprofessional behaviors in the healthcare system as shown in Table 2. The analysis revealed a significant association between gender and unprofessional behaviors, with a 0.007 p-value. No significant relationship was found between the departments in which house officers had worked and their knowledge regarding the meaning and application of professionalism, as indicated by a Chi-square statistic of a p-value of 0.897. The examination of the relationship between the graduating colleges of house officers and their understanding of professionalism yielded no significant findings, with a Chi-square statistic (p= 0.052).

**Table 2:** Chi-square analysis of associations between demographic variables and unprofessional behaviors among house officers. * p-values less than 0.05 was significant.

Variables analyzed	χ²	Sample size (N)	P-value
Gender vs. unprofessional behaviors	3	212	0.007*
Departments vs. knowledge of professionalism	4	212	0.897
Graduating colleges vs. knowledge of professionalism	1	212	0.052*

## Discussion

A survey assessing unprofessional behavior among medical students from year 1 to year 5 revealed that the majority of pre-clinical students perceived unprofessionalism as prevalent. The study highlighted the significance of professional behavior training and the need for a strategic evaluation of the implementation of these training modules [13]. 

The normalization of unprofessional behavior among medical students has contributed to an increased perception of these behaviors as acceptable. This underscores the urgent need for rigorous oversight of unprofessional activities in healthcare settings and for establishing professionalism through accessible training for medical students and professionals. Evaluating the effectiveness of these training programs within their medical environments is critical [14]. 

A study emphasizing the importance of interprofessional communication indicates that enhanced interprofessional education can help students develop a dual identity, fostering collaboration and improving patient care. Training in interprofessional communication skills is essential for addressing ineffective communication and non-respectful listening, which are key factors in interprofessional dilemmas. Incorporating reflective exercises into curricula can enhance students' ability to critically examine their approaches and increase their confidence in managing future dilemmas [15]. 

Skills such as doctor-patient relationship management, reflective practice, time management, and inter-professional relationship skills are crucial for both faculty members and patients. Furthermore, new sub-domains like "effective patient communication" and "demonstrating collegiality" have been identified as significant contributors to medical professionalism in Singapore. Future research in Asian countries should consider incorporating these sub-domains when assessing medical professionalism [16]. 

In a scoping review exploring the relationship between medical professionalism and mental well-being in undergraduate medical education, emerging evidence suggests that compassion towards oneself and patients can reduce burnout and enhance professional satisfaction. However, mental well-being issues are linked to declines in medical professionalism, demonstrating a notable inverse relationship between empathy and burnout. This presents significant risks to medical students' overall health, learning abilities, and future attitudes toward patient care [17]. 

The findings of our study contribute to the understanding of unprofessional behaviors, particularly regarding the lack of significant associations between certain variables such as department and type of institution. The absence of significant findings in these areas suggests that unprofessional behaviors may not be confined to specific settings or departments, indicating a more pervasive issue within medical education and practice. This insight emphasizes the need for comprehensive and institution-wide strategies to address unprofessional behavior, rather than targeting specific departments or types of institutions [18]. 

Our study offers valuable insights for improving medical students' experiences with professionalism education. We aim to engage with faculty about current strategies for teaching and modeling professionalism at the Arabian Gulf University College of Medicine and Medical Sciences and to explore changes in both formal and informal curricula across basic sciences and clinical years to enhance professionalism education. The results also carry policy implications for faculty recruitment, development, and curriculum reform. We recommend that faculty perform self-assessments of their professionalism teaching and discuss curriculum enhancements to further integrate professionalism.

Strengths and limitations

This study has several strengths. First, the use of a pre-validated and pre-tested questionnaire ensured that the data collection tool was both reliable and accurate in assessing the variables of interest. The Cronbach’s alpha value of 0.83, and test-retest reliability coefficient of 0.85, demonstrated high internal consistency and stability over time. Additionally, by including a large sample of house officers from various public and private hospitals across Pakistan, the study captured a diverse range of experiences and insights into professional and unprofessional behaviors in different clinical settings. This broad sample adds to the generalizability of the findings. The use of an online questionnaire via Google Forms also facilitated easy participation, increased anonymity, and reduced potential biases related to face-to-face data collection methods.

Despite these strengths, the study has limitations that need to be acknowledged. First, the use of convenience sampling may introduce selection bias, as the sample may not represent the entire population of house officers in Pakistan. House officers who had more availability or stronger opinions on professionalism might have been more likely to participate, possibly skewing the results. Second, self-reported data on unprofessional behaviors can be subject to social desirability bias, where participants may underreport their involvement or overreport their familiarity with professional ethics. Additionally, the cross-sectional design only provides a snapshot of the participants' experiences at one point in time, limiting the ability to infer causality or changes over time. Finally, while the study employed Chi-square tests to analyze associations between variables, it did not account for potential confounding factors that might influence the relationship between demographics and professional behavior.

## Conclusions

The study found that a significant majority of house officers (99.1%) were familiar with professional ethics, though 53.8% had not received formal medical ethics training. Unprofessional behaviors such as lack of respect for religious and cultural differences (46.2%, p=0.034), refusal to accept critiques (69.8%, p=0.028), and dishonesty in the workplace (68.4%, p=0.0021) were observed frequently, with medical negligence (71.7%, p=0.0001) being notably high. These findings highlight a gap between theoretical knowledge of professionalism and practical adherence in clinical settings. Future research should explore interventions that enhance ethical training and long-term behavioral change among healthcare professionals. Policymakers must integrate comprehensive ethical training into medical curricula to effectively align ethical knowledge with professional practice and mitigate unprofessional behaviors in healthcare settings.

## Appendices

**Table 3:** Questionnaire

Behaviours	Observed	Participated	Observed & participated
Lack of maintaining medical dignity in their relationship, talking, dressing			
Lack of respect for people’s religious and cultural differences			
Lack of self-assessment and refusal to accept and apply constructive critiques			
Lack of commitment to continuous learning			
Lack of equity and fairness in serving patients			
Lack of acceptance of probable health risks him/herself in front of the patient’s			
Failure to comply with hospital regulations and policy			
The lack of bearing difficulty and discomfort in responding to the medical needs of the patients			
Disregard educational activities (e.g., arriving late to rounds for nonclinical reasons, skipping a lecture or seminars in which attendance is required			
Denial of any errors, mistakes and wrongdoing			
Medical negligence in duties in the hospital setting			
Dishonest behavior in the workplace			
Lack of observance of discipline in medical work			
Lack of commitment to be available and responsive when “on call”			
Prefer their interests to the interests of the patient			
Not suggesting treatment options to patients who cannot afford them			
Lack of commitment to patient privacy			
Lack of commitment to privacy of the patient-physician relationship			
Failure to perform duties in teamwork			
Play down feelings, needs and wishes of the patient			
Failure to maintain a professional boundary in relation to patients or colleagues			
The use of alcohol or drugs in the workplace			
Addressing patient inappropriately			
Lack of empathy and compassion with patients			
Failure to report the risky and/or inappropriate behavior of a colleague. (after approaching the individual)			
Failure to introduce yourself and nurses and physician assistants to the patient and his family			
Performing procedures without having sufficient skills (without supervision)			
Having personal conversations or making fun of students, other physicians, peers, or staff in the corridors of the hospital			
Eating or drinking in the hallway of the hospital			
